# Effects of Emotional Context on Memory for Details: The Role of Attention

**DOI:** 10.1371/journal.pone.0077405

**Published:** 2013-10-07

**Authors:** Johann Sung-Cheul Kim, Gerhard Vossel, Matthias Gamer

**Affiliations:** 1 Department of Systems Neuroscience, University Medical Center, Hamburg-Eppendorf, Germany; 2 Department of Psychology, Interdisciplinary Research Group Forensic Psychophysiology, Johannes Gutenberg-University, Mainz, Germany; The University of Queensland, Australia

## Abstract

It was repeatedly demonstrated that a negative emotional context enhances memory for central details while impairing memory for peripheral information. This trade-off effect is assumed to result from attentional processes: a negative context seems to narrow attention to central information at the expense of more peripheral details, thus causing the differential effects in memory. However, this explanation has rarely been tested and previous findings were partly inconclusive. For the present experiment 13 negative and 13 neutral naturalistic, thematically driven picture stories were constructed to test the trade-off effect in an ecologically more valid setting as compared to previous studies. During an incidental encoding phase, eye movements were recorded as an index of overt attention. In a subsequent recognition phase, memory for central and peripheral details occurring in the picture stories was tested. Explicit affective ratings and autonomic responses validated the induction of emotion during encoding. Consistent with the emotional trade-off effect on memory, encoding context differentially affected recognition of central and peripheral details. However, contrary to the common assumption, the emotional trade-off effect on memory was not mediated by attentional processes. By contrast, results suggest that the relevance of attentional processing for later recognition memory depends on the centrality of information and the emotional context but not their interaction. Thus, central information was remembered well even when fixated very briefly whereas memory for peripheral information depended more on overt attention at encoding. Moreover, the influence of overt attention on memory for central and peripheral details seems to be much lower for an arousing as compared to a neutral context.

## Introduction

In daily life we frequently experience that emotional events can be vividly remembered for years. Studies on such highly emotional real-life events (e.g., witnesses to crimes, trauma, flashbulb memories) have shown that strong negative experiences are very well retained over time. However, high accuracy of these memories seems to be limited to information directly associated with the negative event itself (for a review, see [1]). Findings from laboratory studies also showed that negative emotional material is more likely to be remembered than neutral stimulus material. This emotional enhancement effect on memory has been demonstrated using diverse stimulus material, such as pictures [2], words [3] or films [4]. However, studies also found the opposite effect, with negative stimulus material leading to a reduced memory performance when compared to neutral material [5,6]. To resolve this discrepancy, Christianson [1] suggested to post-hoc differentiate results of previous studies into those regarding more central and those regarding more peripheral to-be-remembered details. With this differentiation an emotional enhancement effect on memory similar to real-life events seems to exist, but only regarding memory for more central details. Furthermore the studies reviewed indicated that this benefit in memory for central details comes at the expense of memory for more peripheral details.

This hypothesis of an emotional trade-off effect on memory for central vs. peripheral information is also substantiated by findings of applied research on eyewitness testimony. For example, the presence of a weapon in a crime seems to have a negative effect on the ability to identify the perpetrator (a peripheral detail), while the weapon itself (the central detail) is remembered well. This effect is referred to as “weapon-focus” effect [7] and a meta-analytic review [8] confirmed the presence of this effect across different experimental settings (e.g., “real life” enactments, videos, slide presentations). Interestingly, the overall effect size was comparably small for lineup identification (Cohen’s d = 0.13), but the effect size for more peripheral information (e.g., other characteristics of the perpetrator, like his clothing) was higher (d = 0.55).

To investigate the conceptual distinction of central vs. peripheral information in emotional memory, Burke, Heuer and Reisberg [9] assessed memory for information of different categories after participants viewed a picture story either in a negative arousing version or a neutral one. In line with the assumption of differential effects on memory for central vs. peripheral details, Burke, Heuer and Reisberg found that memory for plot-relevant information was enhanced in the negative emotional condition. Also memory for those plot-irrelevant information that was temporally or spatially associated with the central figures was higher in the emotional compared to the neutral condition. On the other hand, memory for information that was plot-irrelevant and temporally or spatially not associated with the central figure was undermined.

Similar to this experiment, several early studies on memory for details had a relatively strong focus on ecological validity. The stimuli consisted of short films (e.g., [6,10]) or picture stories (e.g., [9,11,12]) that illustrated a storyline within natural complex environments. Results of these studies, however, were inconclusive regarding the effect a negative context has on memory e.g., [5,6,12-14]. More recent laboratory studies did not specifically focus on ecological validity but more on experimental control. Most of these studies used a set of unrelated stimuli depicting diverse natural scenes (e.g., from the International Affective Picture System [15]). And these more recent, scene driven studies furthermore differed from the thematically driven early research, in that the central test-item was the picture itself [16] or the central one-third of the picture area [17] or a letter superimposed on the stimulus material for a short time [18]. Peripheral test-items were objects that were placed outside-around the pictures [16], the peripheral two-third of the picture area [17] or digits presented around the pictures [18], respectively. Other studies (e.g., [19]) used neutral background material (e.g., a street) as the peripheral information, on which they artificially superimposed objects, that were the central (negative vs. neutral) to-be-remembered content (e.g., a car vs. an accident-car), using image-editing software. These more recent laboratory studies found support for the trade-off effect and thus confirmed the hypothesis that emotions have a differential effect on memory for central vs. peripheral details. However, it is unclear whether the results of more recent, scene driven studies − that were conducted using relatively artificial stimulus material and thus also applied a relatively artificial (but more controlled) conception of centrality − generalize to rich sensory experiences of complex events in real life.

Ecological validity or generalizability of experimental findings can be severely limited by non-natural, artificially restricted stimuli. Alan Kingstone [20] for instance pointed out that gaze fixations on social stimuli can strongly depend on further contextual input (e.g., viewing behavior to a cutout face vs. viewing behavior to a face presented in a full-body picture). A similar argument was made by Zaki and Ochsner [21], showing neuroscientific conclusions that crucially depended on studies implementing a methodologically progressive approach to quantify social cognition by using a more naturalistic, video based context. In research investigating the processing of “nonmoving” picture stimuli a different but somehow related insight was proclaimed: The necessity to understand sequential image comprehension. Cohn, Paczynski, Jackendoff, Holcomb and Kuperberg [22] investigated sequential images and found that comprehension requires “the combination of meaning (semantic relatedness) and structure (narrative structure/syntax) to build context across a sequence” (p. 35). Especially when considering, that findings of early, ecologically more valid studies on emotional memory were only post-hoc consistent with the assumption of the trade-off effect e.g., [5,6,12-14], it seems difficult to estimate the generalizability of the emotional trade-off effect on memory for central vs. peripheral information. Additionally, in real life a distinction of central and peripheral information is not clear-cut but a question of continuum. It is challenging to define centrality or importance of information in reasonably general terms and still preserve specificity regarding the emotional trade-off effect on memory (for reviews, see [1,23,24]). In conclusion it seems desirable to verify the emotional trade-off effect on memory using ecologically more valid stimuli and test material. Moreover, instead of relying on only one picture story e.g., [9,11,12,25], it seems necessary to use a larger pool of ecologically more valid stimuli to test for the generalizability of the emotional trade-off effect on memory.

Another focus of this study aimed to investigate the causes of the emotional trade-off effect on memory for central vs. peripheral information. It is generally assumed, with reference to Easterbrook [26], that negative emotional stimuli narrow attention to central information, which in turn enhances memory for these details and reduces memory for peripheral information (for reviews, see [1,23,24]). However, only a few studies directly examined the hypothesis that attentional processes underlie the trade-off effect on memory by measuring or manipulating overt attention and these studies provided inconsistent results. Loftus, Loftus and Messo [7] found that attention to the central detail of a picture story was enhanced in the negative emotional (vs. neutral) condition, but memory for the central information was comparable between both conditions. In line with the emotional trade-off effect, memory for more peripheral details seemed to be diminished in the negative condition; however, attention to the peripheral details was not investigated in this study. Wessel, Van Der Kooy and Merckelbach [14] found more overt attention for central items and less for peripheral items in a negative, compared to a neutral condition. However, in this study the accordant differential recall-pattern did not occur; that is, participants in the emotional group did not show enhanced memory for central information, nor did they display impaired memory for peripheral information. More interestingly, Christianson, Loftus, Hoffman and Loftus [11] controlled attention of participants by showing a fixation cross directly before stimulus onset at the position of the subsequently presented central detail. The stimuli were shown for only 180 ms to prevent eye movements to other picture parts. Although attention was controlled by this means, participants remembered the central detail in the emotional negative condition better than in a neutral condition. In a methodologically somewhat different approach, Riggs, Mcquiggan, Farb, Anderson and Ryan [16] found evidence in support for the emotional trade-off effect in both, attention and memory. But importantly, attention only partially mediated memory enhancement for centrally presented negative pictures and it did not explain reduced memory for peripheral information. In a more recent study, Steinmetz and Kensinger [27] also reported findings questioning the assumption that visual attention is causing the emotional trade-off effect on memory. Participants showed better “selective” memory for emotional items (i.e., concurrent forgetting of the associated background image). However, fixations were not increased on emotional vs. neutral items. Taken together, the results of these studies do not show conclusive evidence and rather question the common assumption that the emotional trade-off effect on memory is mediated by overt attention.

To examine the role of attention for the emotional trade-off effect on memory in an ecologically more valid but standardized setup, we created a set of 26 picture stories with either an emotionally negative or a neutral context. In each story one central and one peripheral detail was naturally embedded in the scenes. The spatial distance of central and peripheral objects between the two contexts were controlled, thus centrality of objects was primarily defined by the relevance objects had for the depicted storyline. The object that was most closely related to the story always served as the central item, while an object that was also present in the scene but irrelevant for the plot was chosen as peripheral item. Explicit memory for these details was tested in a surprise recognition test and was expected to show the trade-off effect of emotion on memory. Eye movements were acquired to examine the relationship between overt attention during encoding and later recognition memory and explicit affective ratings and autonomic responses were measured to validate the induction of emotional arousal. Hierarchical logistic regression analysis was applied to further elucidate the relationship between arousal and attention at encoding and later memory for details.

## Methods

### Ethics statement

This study was approved by the local ethics committee of the Medical Association, Hamburg, Germany and conducted according to the principles expressed in the Declaration of Helsinki.

### Participants

Sixty-five male subjects, most of them students from various faculties (89%), with normal or corrected to normal visual acuity participated in the study. Four participants were excluded from data analysis, two due to difficulties in obtaining stable eye tracking data and two due to technical problems. The final sample (*N* = 61) had a mean age of 26.3 years (*SD* = 3.7 years).

We examined only male participants because of two reasons: Firstly, to maximize comparability between the emotional conditions, all picture stories were shot from the perspective of a male protagonist. Thus both, the kind of stories presented and the sex of the protagonist could have differential effects on male vs. female individuals. Secondly we aimed to verify the emotional manipulation not only by explicit ratings but also using physiological measurements. Since findings of studies are inconclusive regarding sex differences in physiological responses to emotional stimuli [28], we decided to examine a homogeneous sample consisting only of males.

Participants gave written informed consent and were paid for participation. After the experiment, they completed the Beck Depression Inventory, BDI [29] and the trait version of the Spielberger State Trait Anxiety Inventory, STAI [30]. The BDI scores ranged from 0 to 27 (*M* = 4.73, *SD* = 4.50) and the STAI scores ranged from 21 to 53 (*M* = 33.97, *SD* = 6.17). The STAI scores were well comparable to published norms for this age group (*M* = 33, *SD* = 10). Additionally, all analyses were also rerun excluding one participant who had a BDI score greater 13 (i.e., 27), the cut-off value for no to minimal depression, and another one who had a STAI score higher than the 90^th^ percentile of the norm sample (i.e., 53). Results for this restricted sample were very similar as compared to results based on all participants.

### Stimuli

#### Picture stories

**Figure 1:**
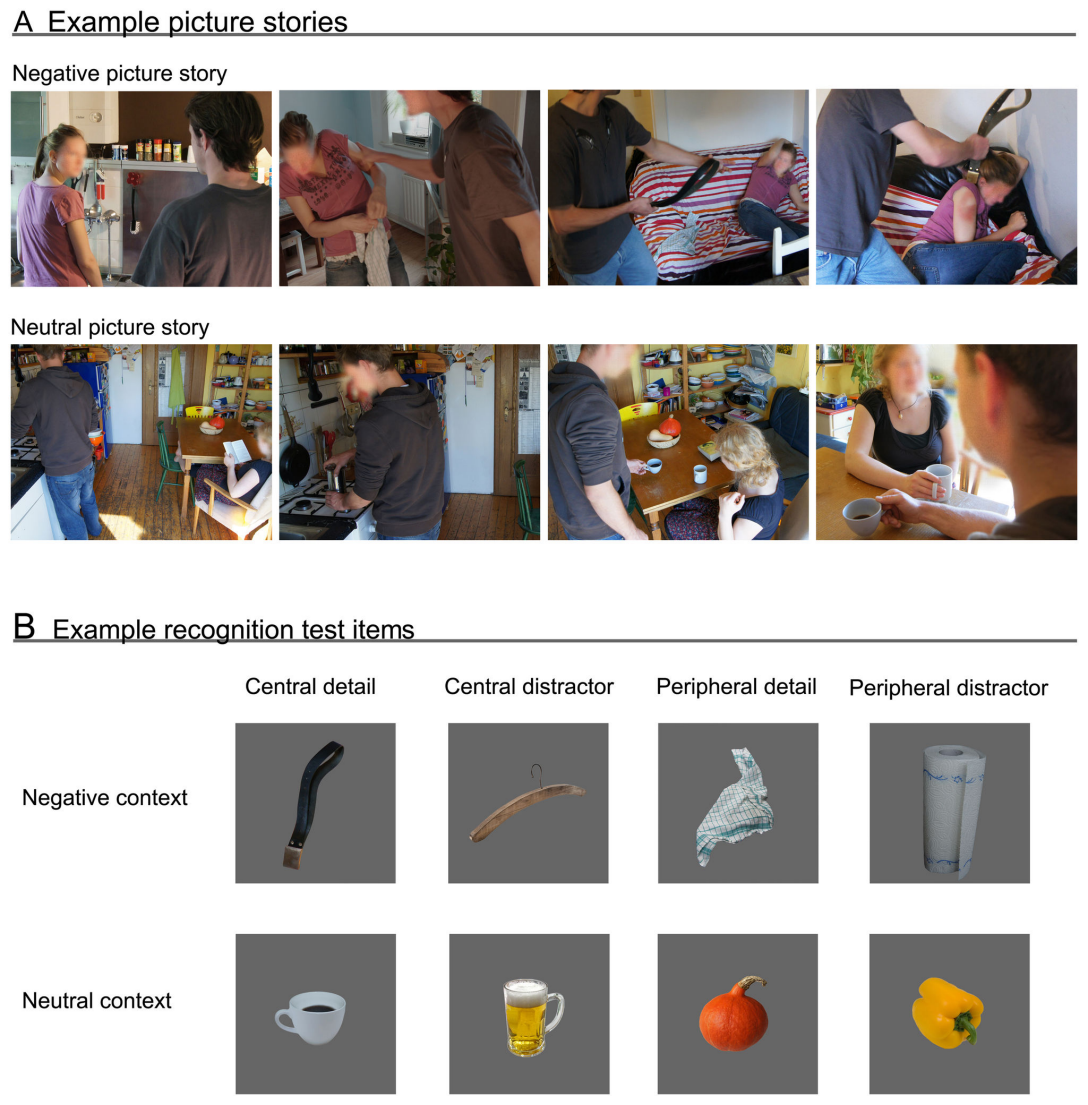
Example stimuli. Negative and neutral example stories of the encoding phase (A), negative and neutral example items of the surprise recognition test (B). Note, to protect privacy, faces of persons depicted in the example picture stories were blurred for publication, but were clearly visible for participants in the study.

Similar to previous studies, we used picture stories as stimulus material (for example picture stories and test items, see Figure 1). However, instead of using only one story (e.g., [9,11,12,25]), we developed a larger set of stimuli to be able to examine the validity of the emotional trade-off effect on memory across different story contexts. To this aim 13 negative and 13 neutral picture stories were shot with each story consisting of 4 pictures with a resolution of 1600 x 1200 pixels. While the first picture introduced the setting and was always of neutral valence, from the second picture on negative stories illustrated incidents such as domestic violence, vandalism, burglary, a fight or a murder. The negative stories varied in the severity of the event but all depicted a plot that could be a matter of criminal law in real life. Neutral stories only depicted amateur actors in daily non-emotional activities, such as a couple having a cup of coffee, someone buying something in a shop, or a person eating an apple (see Table S1 for a complete list of the stories).

All stories were shot from the perspective of a male protagonist. Negative and neutral stories were balanced in regard to the number and sex of the persons involved and to the kind of location the story took place at (i.e., for each negative story involving a male protagonist and a female victim a neutral story was constructed that took place at a similar location and also involved a male and a female actor). In each picture story two relevant objects were naturally embedded in the scenes for a subsequent recognition test. One object was of central relevance for the plot while the other one was peripheral and irrelevant for the storyline. The object that was most closely related to the story always served as the central item (e.g., a belt someone used to beat up a woman or a cup of coffee someone serves a woman). An object that was also present in the scene but irrelevant for the plot was chosen as peripheral item (e.g., a dish towel in the middle-ground of a domestic violence scene or a pumpkin on the table at which a couple was sitting). Both, the central and the peripheral object appeared clearly visible in two of the 4 pictures of a story and the picture number in which central and peripheral items occurred in (i.e., 1^st^ to 4^th^ picture) was balanced across contexts. None of the objects nor objects of a same category appeared in any of the other stories again. Most of the central test items and all peripheral test items were technically neutral regardless of the emotional context. However, 4 out of 26 central items can be considered to be arousing themselves (e.g., a knife) but these items were balanced between the emotional and the neutral context.

We also controlled for the absolute spatial distance of items from the center of the pictures and the ROI sizes. Central items of negative picture stories had an average distance of 296 pixels from the screen center, which did not differ significantly from the neutral picture stories (*M* = 293 pixels distance), *t*(50) = 0.07, *p* = .94. Also the distance of peripheral items from the image center did not differ significantly between contexts (negative: *M* = 407, neutral: *M* = 491, *t*(50) = 1.59, *p* = .12). Importantly the difference of distances between central and peripheral items did not differ between negative and neutral picture stories, *t*(50) = 1.20, *p* = .24. Furthermore, none of the comparisons regarding the ROI sizes measured in pixels were significant (central negative: *M* = 56541, central neutral: *M* = 74933, *t*(50) = 0.68, *p* = .50; peripheral negative: *M* = 36918, peripheral neutral: *M* = 42727, *t*(50) = 0.49, *p* = .63; negative [central – peripheral] vs. neutral [central – peripheral], *t*(50) = 0.43, *p* = .67).

#### Recognition test items

A photo of each central and peripheral item was taken individually for the surprise recognition test. Care was taken to portray each object from a similar perspective as they had appeared in the picture stories. Further, for each central and peripheral target item, three distractor test items were constructed. We decided to construct a larger set of distractor items to be able to use the same stimulus set in future studies involving the presentation of multiple distractors for each target item (e.g., studies using variants of the Concealed Information Test [31]). However, since all results did not differ significantly between distractor sets, we decided to pool the data across sets for all statistical analyses. Distractor items were developed based on the following 3 criteria: First, a distractor object had to be as plausibly to appear in the story as the respective target item. Second, the picture of the distractor item was taken from a comparable perspective as the corresponding target item. Third, none of the distractor objects nor objects of a same category appeared in any of the other stories. With 13 negative and 13 neutral picture stories, each containing a central and a peripheral object, there were a total of 52 target items and 156 distractor items. The distractor items were divided into three sets, each containing one distractor item for every target item. For each participant 52 target items and 52 items of one distractor set were used in the recognition test. The distractor sets were counterbalanced across participants.

### Apparatus

Physiological responses were recorded with a Biopac MP100 device (Biopac Systems, Inc.). Skin conductance was measured at the thenar and hypothenar eminences of the participant’s non-dominant hand by a constant voltage system (0.5 V) using a bipolar recording with two Hellige Ag/AgCl electrodes (surface area = 1 cm^2^) filled with 0.05 M NaCl electrolyte. An electrocardiogram (ECG) was recorded using 3M RedDot Ag/AgCl electrodes filled with electrode paste and attached to the manubrium sterni and the left lower rib cage, the reference electrode was placed at the right lower rib cage. Eye movements were monitored using a video-based eye-tracker (EyeLink 1000, SR Research, Ontario, Canada) with a spatial resolution of less than 0.01° and a spatial accuracy of 0.25°-0.4° and were recorded during the encoding phase with a sampling rate of 1000 Hz. The head location was fixed using a chin rest and a forehead bar. The Software Presentation (Neurobehavioral Systems) was used to present the stimuli on a 19” LCD monitor and to automate the recordings of the eye tracking and the physiological data. Participants viewed the screen from a distance of 47 cm, and responded by using a standard keyboard. Measurements were conducted in a sound-attenuated room, all recording and programming equipment was located outside the room.

### Procedure

Upon individual arrival at the laboratory, participants were told that they would be shown a set of picture stories and that the purpose of the experiment was to quantify the experience, physiological responses and visual interest when viewing these picture stories. They were told that the stories had been constructed for this purpose and asked to watch the pictures closely and to try to get engaged with the storyline. There was no indication that memory for objects appearing in the stories or memory for the stories themselves would be tested later. After applying the electrodes, participants were seated and the chin rest and forehead bar were adjusted. To get used to the valence and arousal rating-scales that were employed for measuring the affective quality of the picture stories, participants were given 6 practice trials each consisting of one picture. Pictures for the practice trials were collected from the Internet and depicted a scene with negative (3) or neutral (3) content. After adjustment of the eye-tracking camera, a 9-point calibration procedure was completed before presenting the picture stories. Each story started with a central fixation cross, shown for 6000 ms and an additional 0-2000 ms for a random delay. Participants were instructed to look at the fixation cross. Subsequently, the 4 pictures of a story were presented consecutively, each with a duration of 10 seconds and a size of 47.1° by 36.5° of visual angle. Picture presentation was terminated with another fixation cross lasting for 2000 ms. After every picture story, participants rated their emotional experience on a computerized version of the Self-Assessment Manikin (SAM; [32]), a non-verbal self-report measure consisting of two bipolar nine-point scales representing the affective dimensions valence and arousal. The eye-tracker was recalibrated after half of the trials and the order of the stories was randomly chosen for each participant. Picture stories were presented without any additional information, neither a title nor a narrative was given. Upon completion of the encoding session participants performed a filler task completely unrelated to this experiment. Instruction and performance of the filler task took approximately 10 minutes. Then participants were instructed for the surprise recognition task. Objects were presented sequentially and participants were asked to indicate whether or not they had seen them in the picture stories before by pressing corresponding keys on a computer keyboard. The instruction emphasized on the correctness of the response instead of the response speed. Target and distractor items were presented in random order with a size of 18.6° by 18.6° of visual angle and a duration lasting until a response was given. Afterwards a fixation cross was shown during the intertrial interval that varied randomly between 5000 ms and 7000 ms.

### Data Processing

#### Heart Rate

To quantify phasic stimulus related heart rate (HR) changes, R-waves were detected from the ECG data and R–R intervals were converted to HR (in beats per minute). Afterwards a real time scaling procedure was applied [33] resulting in one HR value for each of the 40 seconds post picture story onset. The HR in the last second prior to story onset represented the prestimulus baseline. Poststimulus difference scores (∆HR) were derived by subtracting the prestimulus baseline value from the HR-score of each poststimulus second.

#### Electrodermal Responses

Two measures were derived from the electrodermal recordings: Changes in the skin conductance level (SCL) and the number of nonspecific skin conductance responses (#NSRs) during story presentation. For SCL quantification, skin conductance recordings were low pass filtered using a cutoff frequency of 0.05 Hz. Afterwards the mean SCL value was obtained for each picture. The mean SCL of the last second prior the onset of each story represented the baseline and was subtracted from the SCL values of the 4 pictures of each story. The number of nonspecific skin conductance responses was determined for each picture of the stories by counting all responses occurring at least 1 sec after picture story onset. Skin conductance responses were required to exceed an amplitude of 0.02 µS.

*Eye Movement Data* were parsed into saccades and fixations using Eyelink’s standard parser configuration, which classifies an eye movement as a saccade when it exceeds 30°/sec velocity or 8000°/sec^2^ acceleration. Subsequently x and y coordinates of fixations were drift corrected with reference to the central fixation cross at the start of each trial. For each central and peripheral item an outline was drawn around the region of interest (ROI). Fixations were attributed to a target item when they were within the region’s pixel coordinates. The first fixation that occurred after each picture onset was removed from the data to eliminate confounding effects of participants’ previous attentional focus (e.g., fixation cross, fixation from a previous picture).

Two attentional measures for central and peripheral items were derived from the eye movement recordings: The latency of the first fixation on the ROI measuring how fast a ROI was fixated for the first time after stimulus onset and the proportion of viewing-time spent on the ROI relative to the total fixation time during picture presentation (excluding blinks and saccades).

### Statistical analyses

All statistical analysis were accomplished with R, an open-source language for statistical computing (www.r-project.org). Parameter estimations and model fit evaluations for the hierarchical regression analyses were done using the R-package lme4 [34]. An a priori significance threshold of α = .05 was used but marginally significant effects (*p* < .10) are also reported. Cohen’s *d* and *f* are depicted as effect sizes for pair wise comparisons and analyses of variance (ANOVAs), respectively.

#### Affective ratings

To compare the affective quality of negative and neutral picture stories, mean subjective ratings of valence and arousal were each analyzed by paired *t*-tests (negative vs. neutral).

*Physiological responses* between negative and neutral picture stories were compared using a series of 2 x 4 repeated measures ANOVAs with emotional context (negative, neutral) and picture number as within-subject factors.

#### Eye movement data

The latency of the first fixation and the proportion of viewing time on central and peripheral items were compared between negative and neutral stories in a 2 x 2 repeated measures ANOVA with emotional context (negative, neutral) and centrality (central, peripheral) as within-subject factors. To investigate possible differences in the proportion of viewing across time, a more explorative analysis was run as a 2 x 2 x 3 repeated measures ANOVA with emotional context (negative, neutral), centrality (central, peripheral) and time (1^st^ third, 2^nd^ third, 3^rd^ third of picture presentation times) as within-subject factors.

*Recognition memory* for central and peripheral details was determined by subtracting the proportion of false alarms (erroneous recognition of distractor items) from the proportion of hits (correct recognition of items) and analyzed in a 2 x 2 repeated measures ANOVA with emotional context (negative, neutral) and centrality (central, peripheral) as within-subject factors.

#### Regression analyses

Hierarchical logistic regression analysis was used to examine the influence of affective context, item centrality, and overt attention on recognition memory. Based on individual trial level data, random intercept logistic regression models (e.g., [35]) were estimated to analyze the assumed mediating role of attention for the emotional trade-off effect on memory. In model 1 centrality (i.e., peripheral [-0.5] vs. central [+0.5], mean centered) was entered as predictor of subsequent memory. In model 2.a.1 arousal (i.e., range -3.9 [low arousal] to +4.1 [high arousal], mean centered) was added and in model 2.a.2 the interaction of centrality x arousal was additionally taken into account. In model 2.b.1 analogously the estimates for centrality and proportion of viewing time of details (z-standardized values across all items, range: -0.87 to +5.24) and in model 2.b.2 additionally their interactions were used as predictors. In model 3 all parameters used in the models 2.a.2 and 2.b.2 were entered. Finally, the full model 4 comprised all main and interaction effects of centrality, arousal, and proportion of viewing time.

We decided to restrict the regression analyses to arousal ratings as the measure of affective quality and proportion of viewing time as the index of overt attention since separate analyses using valence (instead of arousal) ratings or the latency of the first fixation (instead of the proportion of viewing time) revealed highly similar results due to substantial intercorrelations between measures (correlation between valence and arousal: *r* = -0.83; correlation between first fixation latency and proportion of viewing time: *r* = -0.44). Additionally, regressions were run with the physiological data but no significant effects were obtained. We furthermore checked whether the results of the regression models involving proportion of viewing time might have been driven by outliers (i.e., values outside M ± 2*SD*) or trials where participants did not look at an item at all. Excluding the latter did not change the pattern of results and excluding both, the trials where items were not fixated at all and outliers (with separate cut-off values for central and peripheral items) did also not alter the pattern of results.

In all models, correct recognitions of items that were depicted in the picture stories were estimated with individual (random) intercepts per subject to account for repeated measurements. All estimated regression coefficients of the predictor variables were modeled as fixed effects, since generalization of the results was emphasized as opposed to analyses of individual differences. Noticeably however, a model with all predictor variables of model 3 but with the coefficients of arousal, centrality and arousal x centrality varying by subject was estimated, too (random intercept random slope model). This model showed nearly identical effects and did not differ significantly from the random intercept only model.

Overall, there were 3172 data points (i.e., 61 subjects x 26 stories x 2 levels of centrality) of memory responses (coded as 0 or 1, respectively). Comparison of model deviances, regression coefficients with their standard errors and average predictive probability are reported. Because of the non linearity of the logistic regression coefficients, a specific difference in one of the predictor variables does not correspond to a constant difference in predicted memory. The average predictive probability is a summary comparable to the linear regression coefficient and gives the expected probability to recognize an item corresponding to a specified value or difference of values in one (or more) of the predictor variables while simultaneously taking into account the varying values of all other (unspecified) predictors. These estimates were logit^-1^ transformed and expressed as percent probability to recognize an item [36], pp. 101 ff.

## Results

### Affective ratings

**Figure 2:**
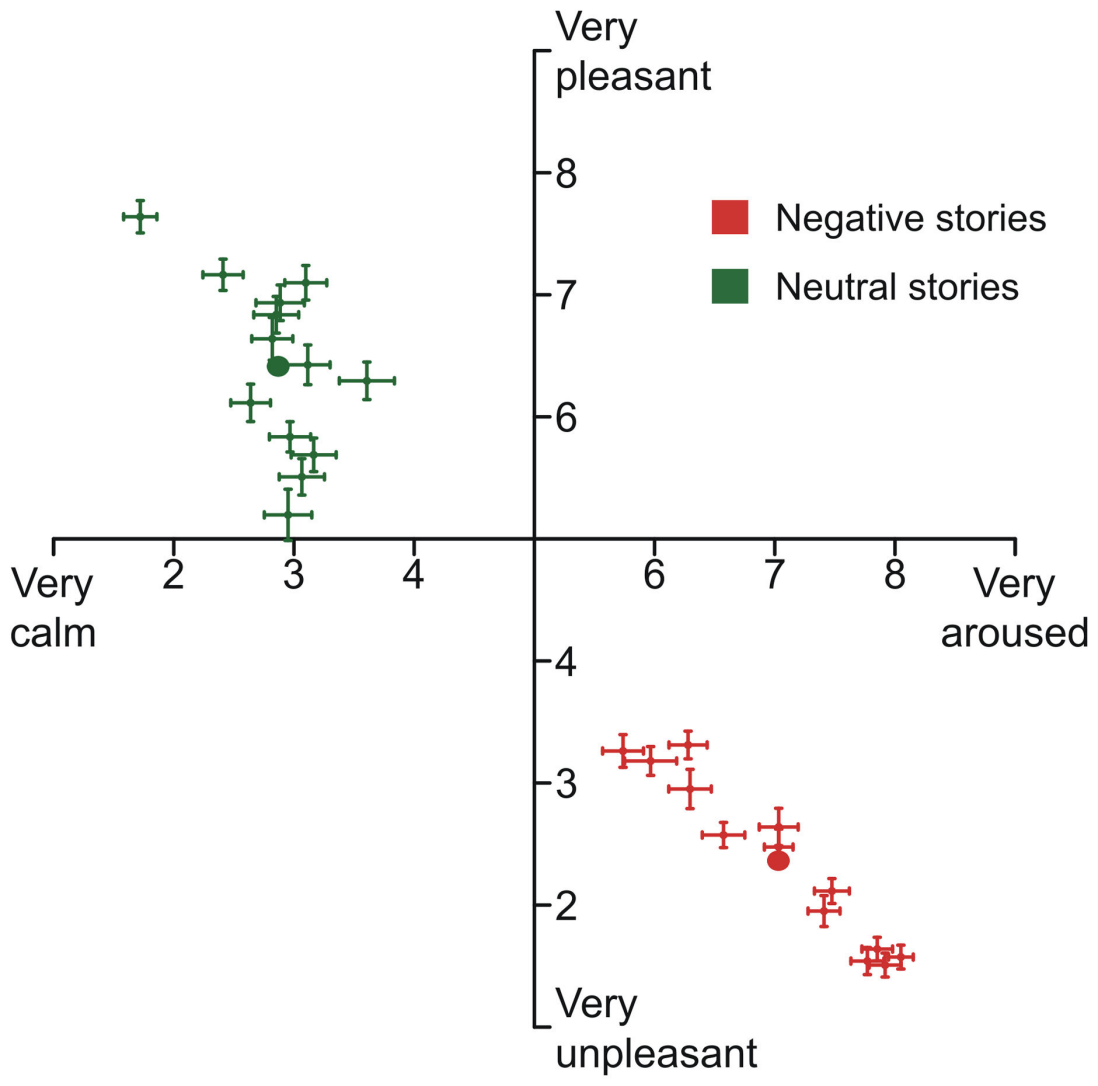
Affective ratings. Mean and standard error of the mean of valence and arousal ratings aggregated per story (crosses); Grand mean and standard error of the mean of valence and arousal ratings for negative and neutral picture stories (filled ellipses).

Consistent with the a priori grouping of negative and neutral stories, results revealed significant differences in valence and arousal ratings between both contexts. Negative picture stories were rated as more unpleasant in valence (*M* = 2.36, *SD* = 0.65), than neutral stories (*M* = 6.41, *SD* = 0.67), *t*(60) = 32.64, *p* < .001, d = 6.08. Also, negative stories were rated as more arousing (*M* = 7.03, *SD* = 0.81), than neutral stories (*M* = 2.87, *SD* = 0.86), *t*(60) = 30.35, *p* < .001, d = 4.98. As depicted in Figure 2, negative and neutral picture stories were clearly separable with respect to the affective ratings.

### Physiology

**Figure 3:**
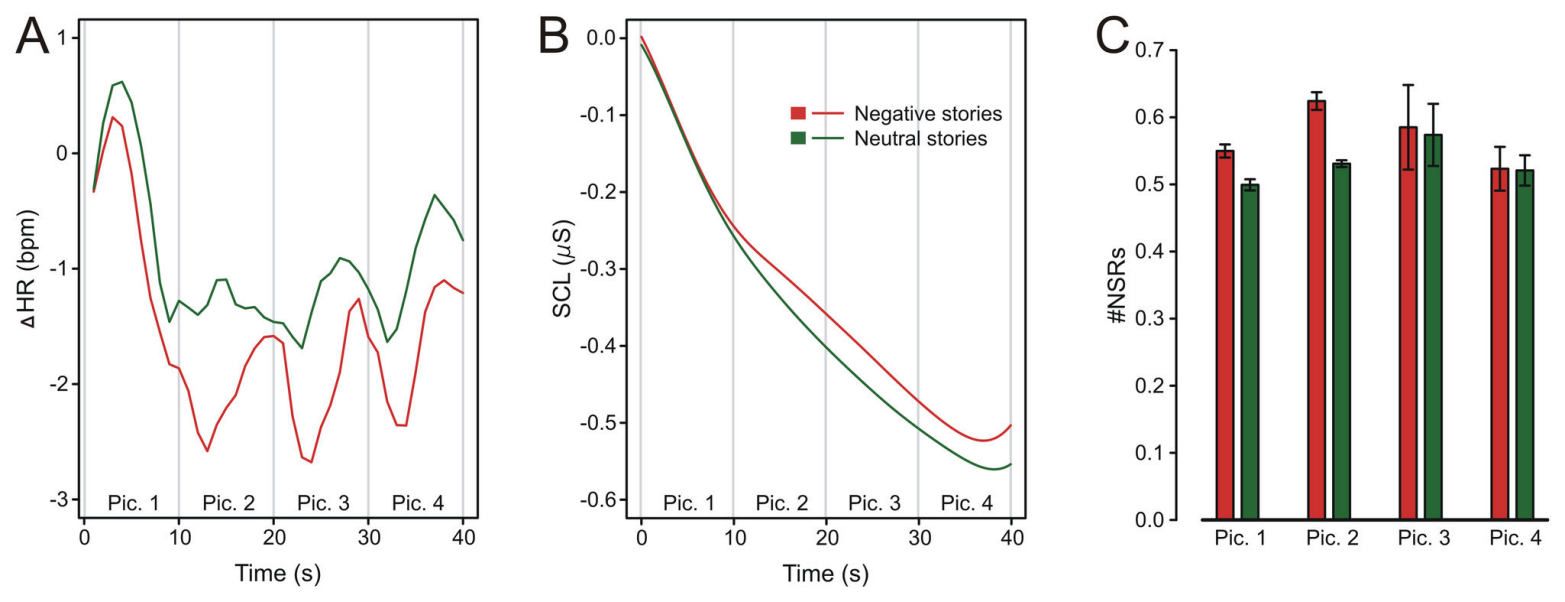
Physiological responses. Phasic heart rate (A), skin conductance level (B) and number of nonspecific skin conductance responses (C) to negative and neutral picture stories as a function of time (picture 1 – picture 4). Error bars indicate standard errors of the mean.

The ANOVA on cardiac responses yielded a main effect of emotional context, *F*(1, 60) = 4.65, *p* < .05, f = 0.13, reflecting a stronger heart rate deceleration for negative picture stories (see Figure 3A). A comparable main effect was neither found for SCL, *F*(1, 60) = 1.82, *p* = .18, f = 0.04, nor for #NSRs, *F*(1, 60) = 2.78, *p* = .10, f = 0.05 (see Figure 3B and 3C). Additionally, the factor picture number was significant for heart rate, *F*(1, 58) = 28.00, *p* < .001, f = 0.18, and for SCL, *F*(1, 58) = 32.92, *p* < .001, f = 0.44, reflecting a general decrease across story presentation. No other main or interaction effect was significant.

### Eye movement data

Regarding latency of the first fixation, the analysis yielded a significant main effect of centrality, *F*(1, 60) = 862.47, *p* < .001, f = 1.43, central items were fixated much earlier than peripheral ones, and a marginally significant main effect of emotional context, *F*(1, 60) = 3.58, *p* = .06, f = 0.07, suggesting that items in negative contexts tended to be attended later. Importantly the interaction of emotional context and centrality was not significant, *F*(1, 60) = 0.00, *p* = .99, f = 0.00.

**Figure 4:**
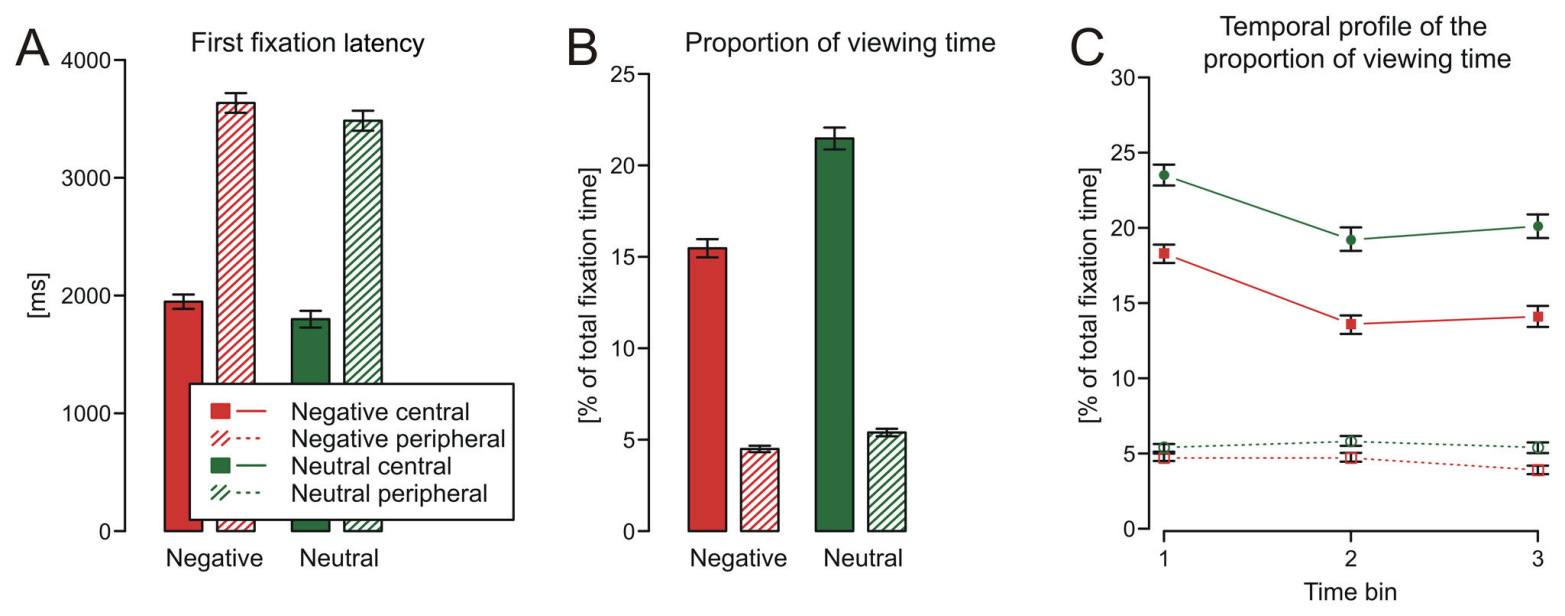
Fixation data. First fixation onset (A), proportion of viewing time (B) and temporal profile of the proportion of viewing time (C) on central and peripheral items in negative and neutral picture stories. Error bars indicate standard errors of the mean.

The ANOVA on the proportion of viewing time also showed a main effect of centrality, *F*(1, 60) = 883.27, *p* < .001, f = 1.76, resulting from a reduced fixation duration on peripheral items. Moreover the significant main effect of emotional context, *F*(1, 60) = 126.12, *p* < .001, f = 0.23, indicates that participants spent less time looking at the items while viewing negative picture stories. Crucially these main effects were qualified by an interaction between emotional context and centrality, *F*(1, 60) = 71.39, *p* < .001, f = 0.17 (see Figure 4B). Though proportion of viewing time of central items was overall higher compared to peripheral items, this difference was much smaller in negative stories, foremost driven by a considerably shorter fixation on central items in negative (*M* = 15.5%, *SD* = 0.5%) compared to neutral stories (*M* = 21.5%, *SD* = 0.6%). The ANOVA on the proportion of viewing time with emotional context, importance and time as independent variables resulted in significant main effects of all three factors. Besides emotional context, *F*(1, 60) = 120.55, *p* < .001, f = 0.21, and importance, *F*(1, 60) = 812.16, *p* < .001, f = 1.36, there was also a significant difference across the three time bins, *F*(2, 59) = 29.39, *p* < .001, f = 0.12. Additionally, the two way interaction importance x time was significant *F*(2, 59) = 28.78, *p* < .001, f = 0.12, while the interaction emotional context x time was not significant. Most interestingly however, the significant interaction emotional context x importance, *F*(1, 60) = 59.33, *p* < .001, f = 0.14, showed a similar pattern as in the ANOVA without the factor time and importantly, the non significant three way interaction indicated no differences in the interaction of context x importance across the three time bins.

### Recognition memory

**Figure 5:**
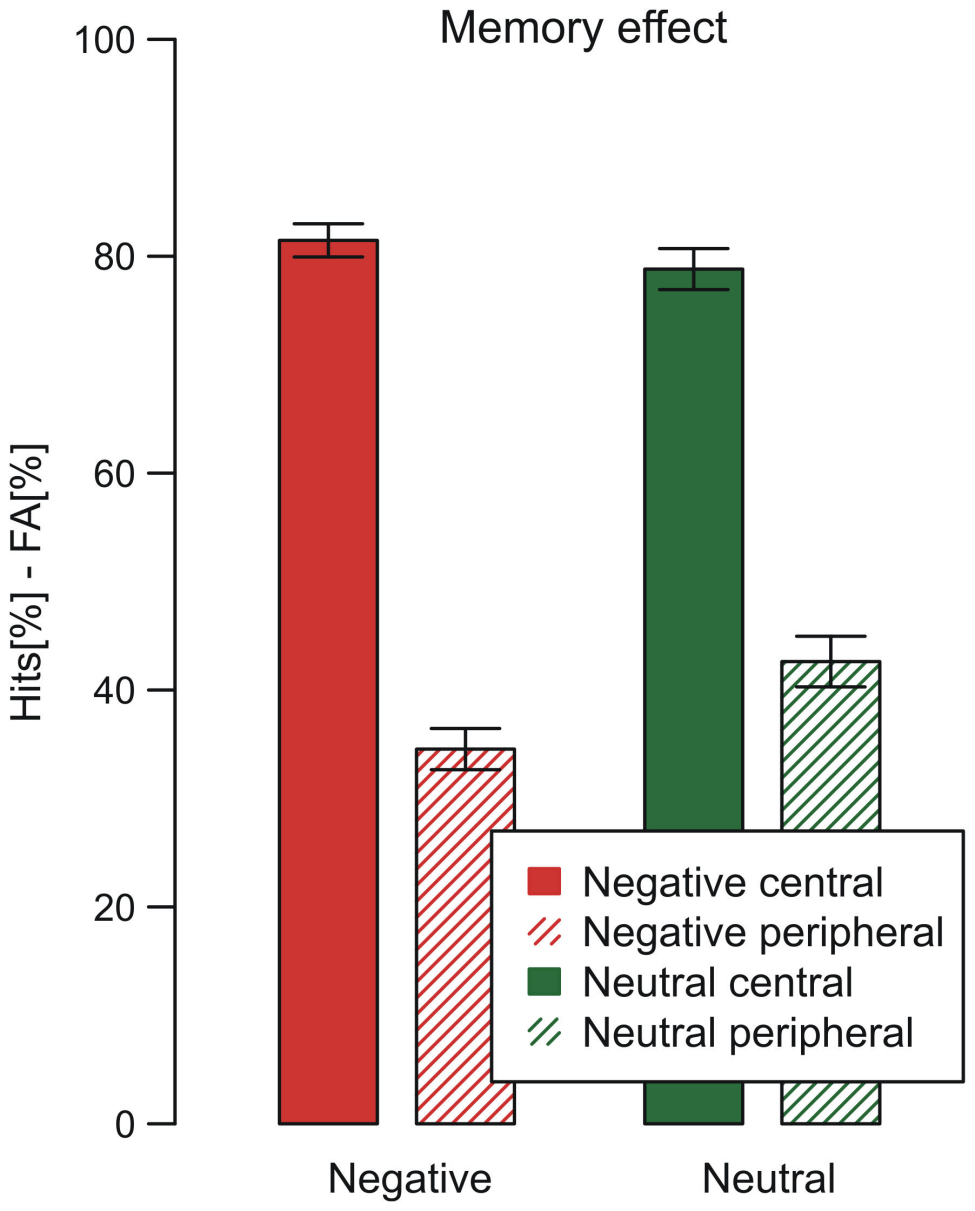
Recognition rates. Mean recognition rate of central and peripheral items occurring in negative and neutral picture stories. Error bars indicate standard errors of the mean.

A significant main effect of centrality, *F*(1, 60) = 643.67, *p* < .001, f = 1.36, showed that central items were remembered more than twice as often as peripheral details. The main effect of emotional context was marginally significant *F*(1, 60) = 3.63, *p* < .10, f = 0.05. Importantly a significant interaction effect was obtained, *F*(1, 60) = 9.25, *p* < .01, f = 0.10, as assumed by the emotional trade-off effect. While recognition for central items (negative: *M* = 0.81, *SD* = 0.12; neutral: *M* = 0.79, *SD* = 0.15) was not modulated by the emotional context, *t*(60) = 1.37, *p* = .17, memory for peripheral items occurring in negative stories (*M* = 0.35, *SD* = 0.15) was considerably worse compared to neutral stories (*M* = 0.43, *SD* = 0.18), *t*(60) = 3.15, *p* < .01, d = 0.49 (see Figure 5).

### Hierarchical regression analyses

In model 1 only centrality was entered and the predicted average difference between recognition of central vs. peripheral items was significant (average predictive probability of central items: 86.3%, of peripheral items: 45.0%, for β*-*values and *p*-values, see Table 1).

**Table 1:** Parameters and deviances of hierarchical logistic regression models predicting recognition memory from centrality, arousal and proportion of viewing time.

	**β**	***SE***	***p***	***AIC***	***BIC***	***Deviance***	***df***
**Model 1**				3457	3475	3451	3
Intercept	0.82	0.07	<.001				
Centrality	2.04	0.09	<.001				
**Model 2.a.1**				3432	3456	3424	4
Intercept	0.83	0.08	<.001				
Centrality	2.06	0.09	<.001				
Arousal	-0.09	0.02	<.001				
**Model 2.a.2**				3428	3459	3418	5
Intercept	0.82	0.08	<.001				
Centrality	2.05	0.09	<.001				
Arousal	-0.07	0.02	<.001				
Centrality x arousal	0.09	0.04	<.05				
**Model 2.b.1**				3304	3328	3296	4
Intercept	0.96	0.08	<.001				
Centrality	1.42	0.10	<.001				
Proportion of viewing time	0.89	0.08	<.001				
**Model 2.b.2**				3236	3266	3226	5
Intercept	1.26	0.09	<.001				
Centrality	0.88	0.13	<.001				
Proportion of viewing time	1.23	0.09	<.001				
Centrality x Proportion of viewing time	-1.49	0.19	<.001				
**Model 3**				3215	3258	3201	7
Intercept	1.25	0.10	<.001				
Centrality	0.90	0.13	<.001				
Arousal	-0.06	0.02	<.01				
Proportion of viewing time	1.22	0.09	<.001				
Centrality x arousal	0.10	0.04	<.01				
Centrality x Proportion of viewing time	-1.46	0.19	<.001				
**Model 4**				3192	3247	3174	9
Intercept	1.26	0.10	<.001				
Centrality	0.91	0.13	<.001				
Arousal	-0.10	0.03	<.001				
Proportion of viewing time	1.22	0.10	<.001				
Centrality x arousal	0.23	0.05	<.001				
Centrality x Proportion of viewing time	-1.45	0.19	<.001				
Arousal x Proportion of viewing time	-0.17	0.04	<.001				
Centrality x arousal x Proportion of viewing time	0.03	0.08	.70				

Note. AIC = Akaike information criterion; BIC = Bayesian information criterion.

In Model 2.a.1 the main effect of arousal was added and (significantly) predicted better memory for items appearing in a low arousing context as compared to items of a high arousing context (see Table 1). That is, objects from a story rated 1 in arousal (mean centered: -3.9) were predicted to be recognized with 78.8% average probability while objects from a story rated 9 (mean centered: +4.1) were predicted to be recognized with 67.2% average probability. The effect of centrality did not change substantially. Model fit statistics showed that the model containing arousal as additional predictor (model 2.a.1) was a significant improvement over the centrality only model (model 1), χ^2^(1) = 26.43, *p* < .001.

**Figure 6:**
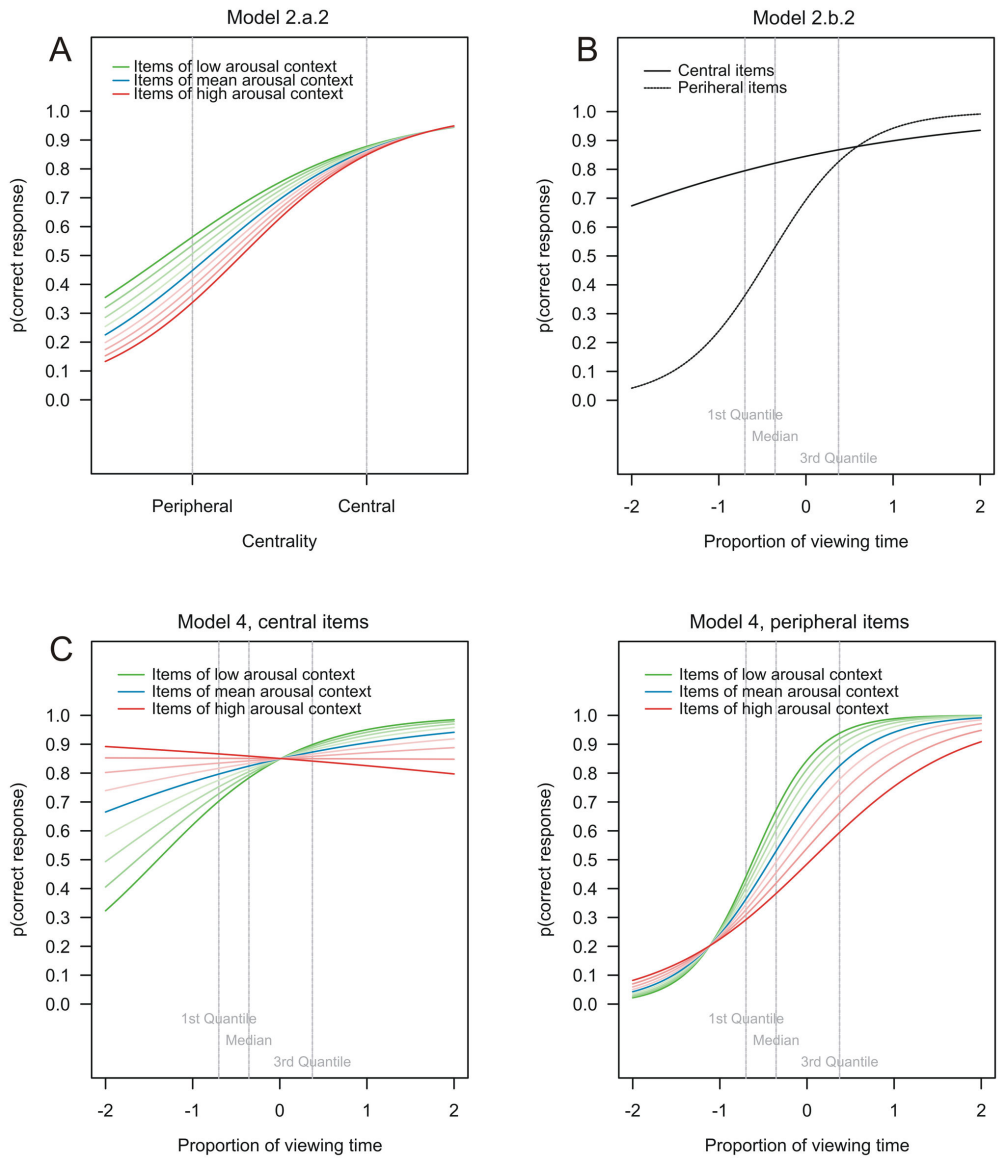
Estimated probabilities of hierarchical logistic regression models. (A) Probability to recognize an item as a function of centrality, for the nine levels of arousal; (B) Probability to recognize a central vs. peripheral item as a function of the proportion of viewing time; (C) Probabilities to recognize a central (left) or a peripheral item (right) as a function of the proportion of viewing time, for the nine levels of arousal.

In model 2.a.2 the interaction effect arousal x centrality was additionally added. The effects of centrality and arousal did not change substantially. Importantly the interaction effect arousal x centrality was significant as well (see Table 1 and Figure 6A). In line with the assumption of the emotional trade-off effect, the estimated model predicted increased differences in memory between central and peripheral items as a function of the arousal level. Central items from a low arousing context (-3.9) were estimated to be remembered with 87.8% probability and corresponding peripheral items with 56.4%, (average difference = 31.4%). By contrast, central items from a high arousing context (+4.1) were estimated with 84.9% and corresponding peripheral items with 33.7% (average difference = 51.2%). Correspondingly, comparison of model deviances suggested that the model including the interaction term arousal x centrality (model 2.a.2) was a significant improvement over the main effects only model (model 2.a.1), χ^2^(1) = 5.83, *p* < .05.

Model 2.b.1 and model 2.b.2 with centrality, proportion of viewing time and their interaction as predictors, showed a divergent pattern. The effect of centrality decreased substantially from model 1 (average difference between central and peripheral items = 41.3%) to model 2.b.1 (average difference = 27.3%), where proportion of viewing time was added as a covariate. Thus, attention seemed to (partly) mediate the differential effect of central vs. peripheral details on memory. The effect of centrality decreased again in model 2.b.2 (average difference = 24.9%), where the interaction effect of proportion of viewing time x centrality was added. Correspondingly and in line with the assumption that proportion of viewing time would affect memory, the main effect of proportion of viewing time turned significant in model 2.b.1 (see Table 1). Across centrality, items fixated for a relatively short duration (1^st^ quartile) were estimated to be recognized with 57.3% average probability and those fixated relatively long (3^rd^ quartile) were estimated with 76.1% probability to be recognized, an average difference of 18.7%. Comparisons of model deviances also suggested that adding the proportion of viewing time (model 2.b.1) was a strong improvement over the centrality only model (model 1), χ^2^(1) = 154.63, *p* < .001. The main effect of proportion of viewing time increased in model 2.b.2. Items with small proportion of viewing time (57.9%) now differed to long durations (84.7%) by an average difference of 26.8%. Crucially the interaction effect was significant as well, suggesting that recognition of central vs. peripheral items are differentially affected by increased proportions of viewing time (Table 1 and Figure 6B). Recognition of peripheral items was predicted to differ strongly dependent on attentional processing. Peripheral items that were fixated shortly (1^st^ quartile) were estimated to be recognized with 36.3%, those fixated for a longer duration (3^rd^ quartile) were estimated with 82.6%. For central items, however, these values were relatively similar and amounted to 79.5% (1^st^ quartile) and 86.8% (3^rd^ quartile), respectively. Correspondingly, adding proportion of viewing time contingent on centrality (model 2.b.2) showed a better fit than the main effects only model (model 2.b.1), χ^2^(1) = 70.42, *p* < .001. Taken together, comparisons of models 1, 2.b.1, and 2.b.2 showed that adding proportion of viewing time and the interaction of proportion of viewing time x centrality as covariates reduced the main effect of centrality thereby substantiating the mediating role of attention on memory for central vs. peripheral items. However, these results also showed that memory for central information depended much less on attentional processes during encoding as compared to memory for peripheral details.

In model 3 all parameters used in the models 2.a.2 and 2.b.2 were entered and comparisons of model deviances suggested model 3 to be an improvement over both models (model 3 vs. model 2.a.2, χ^*2*^(2) = 217.32, *p* < .001; model 3 vs. model 2.b.2, χ^2^(2) = 24.54, *p* < .001). The impact of the proportion of viewing time on memory and the proportion of viewing x centrality did not change substantially in the presence of the main effect of arousal and the interaction effect arousal x centrality (model 3 compared to 2.b.2). The effect of arousal did also not change substantially compared to model 2.a.2. Crucially however the effect of arousal x centrality was not affected by the presence of the covariates involving the proportion of viewing time (see Table 1). Assuming that attention mediates the differential effects of arousal on memory for central vs. peripheral details, a reduced impact of arousal x centrality would have been expected, when controlling for attention. However, instead of a decrease, the impact of arousal x centrality on memory even increased slightly as compared to model 2.a.2.

Interestingly, all effects of model 3 that were related to arousal increased in the presence of the additional predictors arousal x proportion of viewing time and the three way interaction of all factors in model 4. The effect of arousal x centrality even doubled in effect size (see Table 1). All further effects did not change substantially (e.g., centrality, proportion of viewing time, and proportion of viewing time x centrality). Importantly, also the interaction between arousal and proportion of viewing time was significant and moreover, this effect was not significantly different for central vs. peripheral items (see Table 1). The predictive power of the full model was significantly better than for model 3, χ^*2*^(2) = 26.80, *p* < .001. The pattern of results suggests that the amount of overt attention had a higher influence on memory for central and peripheral items appearing in a low arousing as compared to a high arousing context (see Figure 6C). Average predictive probability of a central item from a high arousing context was estimated to be comparably high when fixated shortly (86.6%), or long (84.8%). But for central items from a low arousing context the probability was estimated to be 70.6% if fixated shortly and increased to 88.8%, if fixated longer. Congruently, peripheral objects in a negative context improved much less, with longer fixation durations (1^st^ quartile: 28.9%, 3^rd^ quartile: 59.9%, average difference = 31.0%), as compared to peripheral objects from a more neutral context (1^st^ quartile: 44.4%, 3^rd^ quartile: 93.3%, average difference = 48.9%).

## Discussion

Early research on the effects of emotion on memory had a stronger methodological focus on ecological validity but revealed partly inconclusive results. It was then post-hoc suggested that the centrality of to-be-remembered information plays a crucial role, with central items being remembered better at the expense of memory for peripheral items, when appearing in an emotionally negative context [1]. Though more recent studies confirmed the hypothesis of an emotional trade-off effect on memory, these studies have used more artificial encoding and test material (e.g., [16-19,27]). With a focus on ecological validity, the current study aimed to investigate the emotional trade-off effect on memory for central vs. peripheral details naturally appearing in diverse picture stories. Hypothesizing to find the emotional trade-off effect on memory using this kind of stimuli and test items we furthermore aimed to investigate the causes underlying this trade-off effect. Specifically we were interested in testing the common assumption, that attentional processes are mediating the emotional trade-off effect on memory [1,23,24]. Furthermore we were interested in validating explicit emotional ratings of the stimulus material by physiological parameters and to explore the relationship of physiological reactions during encoding with later recognition memory responses.

The difference found between explicit affective ratings of negative and neutral picture stories validated the a priori groupings of the stories. Picture stories were rated using the same scale and on the same affective dimensions as the IAPS picture set [15,32]. On average, negative picture stories were rated as more unpleasant than 94.4% and as more arousing than 96.7% of the pictures of the complete IAPS set (relative to norms for male subjects), while neutral picture stories were rated on average as more unpleasant than 30.9% and as more arousing than 20.1% of the IAPS pictures.

Differences between negative and neutral picture stories were confirmed by a stronger heart rate deceleration for negative picture stories. This finding is consistent with Burke, Heuer and Reisberg [9], who also used picture stories as stimulus material and physiological measurements for validation and reported a decreased heart rate in the negative (vs. neutral) arousal group. A stronger heart rate deceleration for negative picture stories is also in line with findings of studies using IAPS pictures (e.g., [37]) as stimulus material. Cardiac deceleration in an emotionally negative context seems to be an initial reaction to a threatening stimulus when preparation for an immediate reaction is not required (for a review, see [37]). However the effect size of the heart rate measure was quite small in the current study and additionally we did not observe significant differences in the electrodermal data. These small (heart rate) and non-significant (skin conductance) physiological effects were not expected and indicate that physiological responses seem to depend on specific emotions and methods of induction. Though activity in the autonomic nervous system is an established variable to quantify responses to specific emotions, the pattern of findings is quite inconsistent. Importantly, physiological responses to emotions can be moderated by the method used for emotion-induction and seem to differ strongly for subgroups of negative and positive emotions (for reviews, see [38–41]). For example, other studies using stimuli from the IAPS did only find significantly enhanced electrodermal responding to highly arousing pictures (e.g., mutilation, animal attack, human attack [37]) or to threatening pictures [42] whereas no such response amplification was evident for less arousing negative picture categories, or for victim scenes respectively.

The rather extreme explicit affective ratings obtained in the current study seem to be even more striking. It is important to emphasize, that not isolated pictures were presented in this study but instead sequential picture stories. It could be, that watching a story about a protagonist and often a second person (in the negative condition always a victim) generates more dynamic social cognitions including empathy and affective or moral judgments about the incident or the persons involved. This might have engaged a different subjective involvement of the participants causing these rather extreme ratings of valence and arousal, while strong affective physiological responses remained absent in the ANOVAs and also in the more explorative regressions of affective physiological responses at encoding on subsequent memory.

Memory data from 13 negative and 13 neutral picture stories of the current study confirm the emotional trade-off effect on memory. We observed the expected interaction effect of emotional context x centrality on recognition data. Comparisons of grand means (see Figure 5) indicated, that this effect was mainly driven by a reduced recognition rate for peripheral items presented in a negative (vs. neutral) emotional context and did not suggest an enhancement effect on memory for negative central items. However, the hierarchical regression models allowed for evaluating these effects in more detail. In the full model that incorporated influences of item centrality, arousal, and proportion of viewing time (model 4), the predictive probability of recognizing a central item that appeared in a highly arousing context was larger (85.4%) as compared to a low arousing context (79.8%) and by contrast, an inverse pattern was obtained for peripheral items (high arousal: 46.3% vs. low arousal: 67.4%; see Figure 6 C and D). In conclusion, results from the present study using ecologically more valid stimuli and test items confirm the emotional trade-off effect, that assumes enhanced memory for central information and reduced memory for more peripheral information when appearing in a negative as compared to a neutral context.

To account for the emotional trade-off effect on memory, it is generally assumed, that negative emotional stimuli narrow attention to central information at the expense of attention for peripheral information. This in turn, so the assumption, is the underlying cause for both, enhanced memory of central and for reduced memory of peripheral information in an emotional as compared to a neutral context (for reviews, see [1,23,24]). In the current study, eye tracking was used to test this hypothesis. Interestingly findings with both attentional measures, quantifying how fast and for how long participants attended to the details indicated that items appearing in a negative context were attentionally processed less (i.e. attended to later and for a shorter duration) regardless of centrality. Especially, central items in a negative context were fixated much shorter as compared to a neutral one. Furthermore this result was also evident in an analysis of the temporal profile indicating, that less attentional processing of (even “central”) information in an emotionally negative context happens early and is maintained throughout the whole viewing period. This pattern of fixations did not reflect the memory results and contradicts the assumption that the emotional trade-off effect is mediated by attention. In contrast, these findings suggest, that in a negative context attention for objects is diminished even for central information closely connected to the thematic content, while eye movements seem to be directed more (and earlier) to other information, probably those establishing the emotionality of the negative context itself (e.g., faces / bodies).

Moreover, the hierarchical models also suggest objection of the common hypothesis, that this trade-off effect of emotion on memory is caused by congruent differences in overt attention. Inclusion of fixation data into the regression models did not diminish the joint influence of centrality and arousal. By contrast, the effect sizes of the centrality x arousal term in the two models including attentional parameters as covariates increased partly substantially. Thus a necessary condition for a mediating role of this factor was not met [43], ruling out the possibility that attention is mediating the trade-off effect of emotion on memory (see also [16,27]).

Furthermore, we obtained the following interesting relationships. Firstly, it seems that the effect of attention on memory differs between central and peripheral items. High recognition of objects that are important for the storyline (central items) seems to depend much less on overt attention than recognition of objects irrelevant for the plot (peripheral items). That is, central objects fixated shortly are recognized already relatively good, compared to objects fixated for a longer duration, while peripheral objects are recognized much better only when previously attended to for a longer duration. Secondly, the role of attention for recognition of objects from a high vs. low arousing context differs. Recognition of objects from a more negative context seems to depend less on the amount of overt attention, than recognition of objects from a more neutral context. Such effect was observed for both, central and peripheral details. These results suggest that processing of information of a negative context differs qualitatively from that of a neutral context. For the former, central information seems to be reliably encoded within a few fixations and even enhanced attention does not result in stable memories for peripheral information. In contrast, overt attention is a better predictor for the recognition of central and peripheral details that appeared in a neutral context. Such differential effects might be related to differences in the neural processing of information from an emotionally negative context. Indeed, neuroimaging studies have found reliable evidence, that different brain regions show greater activation when processing emotional as compared to neutral stimuli. For example, processing emotional information is related to increased recruitment of limbic brain circuits including the amygdala e.g., [44,45] and enhancement effects of emotion on memory are related to increased activation of the amygdala, the prefrontal cortex and the mediotemporal lobes (for a review, see [46]). In conclusion, the currently observed reduced effect of attentional processing on subsequent memory for central information of a negative context might indicate a qualitatively different processing of information appearing in a negative context (see also [24]). This hypothesis, however, has to be explicitly tested in future studies using neuroimaging techniques.

Some limitations of this work are also worth mentioning: Firstly, the present study used male participants only, thus generalization of these results to female individuals might be restricted. Secondly, the current hypotheses should be tested using even more advanced stimuli in future studies. Encoding situations that are more similar to real-life experiences should be experimentally simulated. Investigations using controlled video based material or virtual reality technology would be valuable and though measurement of attentional and physiological parameters seems difficult in real life acting situations, studies implementing such technologies seem very promising to ecologically verify and possibly specify in more detail the interacting effects between emotion, attention, and memory.

Taken together, the current results confirm the emotional trade-off effect on memory in an ecologically more valid stimulus set of picture stories, with either of an emotionally negative or a neutral context and a definition of central vs. peripheral details based of their relevance for the storyline. However, our results also indicate a strong objection of the common hypothesis, that the trade-off effect of emotion on memory is caused by corresponding differences in overt attention and moreover imply, that the role of attentional processing for later memory depends on centrality and emotional context but not their interaction.

## Supporting Information

Table S1 **List of all picture stories.**
(DOCX)
