# Supplementary material for: Effects of Emotional Context on Memory for Details: The Role of Attention
Source: PLoS One. 2013 Oct 7;8(10):e77405. doi: 10.1371/journal.pone.0077405 (PMC3792043; doi:10.1371/journal.pone.0077405)
Supplement: Table S1 — List of all picture stories. (DOCX) [file pone.0077405.s001.docx]

| **Negative picture stories** | **Neutral picture stories** |
| --- | --- |
| Robbery (4x) | Eating an apple |
| Vandalism (2x) | Shopping |
| Murder (2x) | Losing gloves |
| Burglary | Chopping wood |
| Kidnapping | Drinking coffee |
| Theft | Opening the door for someone |
| Intimate partner violence | Finding something |
| Affray | Buying a parking ticket |
|  | Putting something in the rear trunk |
|  | Driving the son to the tennis club |
|  | Asking the time |
|  | Pottering about |
|  | Buying cigarettes |
